# Progressive Mitochondrial Encephalopathy Due to the Novel Compound Heterozygous Variants c.182C>T and c.446A>AG in NARS2: A Case Report

**DOI:** 10.7759/cureus.43969

**Published:** 2023-08-23

**Authors:** Josef Finsterer, Sounira Mehri

**Affiliations:** 1 Neurology, Neurology and Neurophysiology Center, Vienna, AUT; 2 Nutrition-Functional Foods and Vascular Health, Biochemistry Laboratory, Faculty of Medicine of Monastir, Monastir, TUN

**Keywords:** mitochondrial encephalopathy, myoclonus, respiratory chain, spasticity, epilepsy, nars2, mitochondrial

## Abstract

Progressive mitochondrial encephalopathy manifesting as developmental delay, regression, epilepsy, myoclonus, dystonia, and spasticity due to a novel compound heterozygous variant in NARS2 has not been reported.

The patient is a 3.5-year-old female with normal psychomotor development until she experienced her first generalized status epilepticus at 4.5 months of age. After seizure control, generalized myoclonus and psychomotor regression became evident. She suffered from two other epileptic states and seizure control remained inadequate despite the use of multiple anti-seizure drugs. Neurologic examination revealed generalized hypotonia, discoordination, unstable eye contact, drooling, open mouth, myoclonus, periodic torticollis, and ankle contractions. Cerebral MRI revealed hydrocephalus ex vacuo due to diffuse cortical and subcortical atrophy bilaterally and incomplete myelination. Genetic testing at 12 months of age revealed the compound heterozygous variants chr11: 78204182C>T and chr11: 78282446A>AG in NARS2. Despite anti-seizure drugs, mitochondrial cocktail, and cannabidiol, the disease progressed to intractable seizures and severe tetraspasticity.

In summary, this case demonstrates that compound heterozygous variants in NARS2 can phenotypically manifest exclusively in the brain with intractable epilepsy, myoclonus, developmental delay, regression, hypotonia, cerebral atrophy, and hypomyelination, followed by tetraspasticity and dystonia.

## Introduction

NARS2 is a nuclear DNA-related gene located on chromosome 11q14.1 [1]. NARS2 encodes mitochondrial asparaginyl-transfer RNA (tRNA) synthetase, an enzyme responsible for the aminoacylation of cognate tRNAs (by loading them with the asparagine amino acid) and arranging for the correct translation of mitochondrial proteins [2]. NARS2 is widely expressed in humans, for example, in the brain, spiral ganglia and the Corti organ, and the vestibular system [1]. NARS2 variants cause a combined oxidative phosphorylation deficiency-24 (COXPD24) [3]. Biallelic variants in NARS2 cause mitochondrial disorders (MIDs) with a broad phenotypic spectrum [1-18]. The following case report describes a patient with MID due to a novel compound heterozygous variant in NARS2 that phenotypically manifested only in the brain. The aims of the report were to improve the understanding of the phenotypic heterogeneity of NARS2 variants and the pathogenesis of COXPD24 [3].

## Case presentation

The patient is a 3.5-year-old female (height: 104 cm; weight: 16 kg) with non-syndromic MID due to a compound heterozygous variant in NARS2. Her psychomotor development was normal until she developed generalized status epilepticus (SE) at 4.5 months of age. After the termination of SE with diazepam (DP), hydroxy-butyrate (a ketone body), and thiopental (TP), generalized myoclonus began (Tables 1, 2). Electroencephalography (EEG) recorded regional epileptiform discharges across the right frontal and left occipital projections. As an antiepileptic drug (AED), she received valproic acid (VPA), which suppressed seizure activity clinically and on EEG for seven months. Clinical neurological examination at the age of 4.5 months revealed a slight developmental delay and moderate hypotonia. At 10 months, she could not crawl, stay, or walk, but could maintain steady eye contact, cooed, babbled, gestured, became interested in toys, manipulated with hands, followed simple instructions, remembered colors, body parts, and animals, and produced syllables and emotionally colored sounds.

**Table 1:** Patients with mitochondrial disorders due to NARS2 variants reported as of the end of July 2023 AS: Alpers syndrome; BGL: basal ganglia lesions; CB: cortical blindness; ch: compound heterozygote; dCMP: dilated cardiomyopathy; DD: developmental delay; F: female; HA: hypoacusis; hz: homozygous; ID: intellectual disability; LA: lactic acidosis; LS: Leigh syndrome; M: male; nr: not reported; OA: optic atrophy; OP: ophthalmoplegia; PMR: psychomotor regression.

Age	Sex	Variant(s)	Dosage	Phenotype	Reference
34 y	F	c.822G>C	hz	Myopathy, dysarthria, facial weakness, ptosis	[10]
26 y	M	c.822G>C	hz	ID, epilepsy	[10]
16 y	M	c.641C>T	hz	AS (DD, PMR, hypotonia, epilepsy, LA, OA, CB, C. callosum agenesis, hypomyelination, reflux, vomiting, hepatopathy, tubulopathy)	[11]
2 y	M	c.1130dupC, c.836C>T	ch	PMR, epilepsy, cortical atrophy, LA, infantile spasms, dysphagia, hypotonia, microcephaly, CB, reflux, dCMP, vomiting, left ventricular hypertrophy	[11]
15 m	M	c.1142A>G, c.969T>A	ch	LS (HA, myocloni, seizures, LA, organic acids, laryngomalacia, cortical atrophy, C. callosum atrophy	[12]
6 m	M	c.1142A>G, c.969T>A	ch	LS (HA, myocloni, poor feeding, seizures, organic acids), laryngomalacia, cortical atrophy, C. callosum atrophy	[12]
40 y	F	c.637G>T	hz	HA	[12]
45 y	F	c.637G>T	hz	HA	[12]
26 y	M	c.637G>T	hz	HA	[12]
30 y	M	c.637G>T	hz	HA	[12]
8 y	M	c.707T>G, c.594+1G>A	ch	PMR, HA, epilepsy, cerebral atrophy, quadriplegia hypotonia, short stature, microcephaly	[13]
1 y	F	c.707T>G, c.594+1G>A	ch	Dysphagia, PMR, hypotonia, myoclonic epilepsy, HA	[13]
				HA, short stature, microcephaly, LA	
2 y	F	c.151C>T, c.1184T>G	ch	PMR, epilepsy, myocloni, HA, LA, cerebral atrophy	[13]
4 y	M	c.500A>G	hz	Hypotonia, epilepsy, PMR, HA, cerebral atrophy, LA	[13]
3 m	M	c.167A>G, c.631T>A	ch	Epilepsy, cerebral atrophy, left ventricular hypertrophy	[9]
4 m	M	c.167A>G, c.631T>A	ch	HA, epilepsy, cerebral atrophy, white matter lesions	[9]
nr	nr	c.731C>G, c.1351C>T	ch	nr	[14]
6 y	F	c.641C>T	hz	Hypotonia, poor feeding, epilepsy, LA, hepatopathy, OA, OP, ptosis, microcephaly, CB, HA, spasticity, myopathy	[7]
25 y	M	c641C>T	hz	Epilepsy, hypotonia, LA, cerebral atrophy, BGL, poor feeding, hepatopathy, dystonia	[7]
17 y	F	c.545T>A	hz	Ataxia, HA, brachymetatarsalia, epilepsy, hallux, clubs	[15]
28 m	F	c.545T>A	hz	HA, epilepsy, ataxia, PMR	[15]
14 m	M	c.1339G>A, c.83_84del	ch	Cerebral atrophy, white matter lesions, myocloni, epilepsy	[16]
nr	M	c.1300C>T, c.1253G>A	ch	LS	[17]
24 y	F	c.731CG, c.556A>G	ch	LS (epilepsy, hypotonia, DD, PMR, chorea, opisthotonus, LA)	[8]
6 m	M	c.1141A>G, c.1290G>C	ch	Epilepsy, HA, hepatopathy, hypotonia, HA	[18]
3 y	F	c.475C>T, c.649T>G	ch	Diabetes, epilepsy, LA, cerebral atrophy	[6]
1 y	M	c.475C>T, c.649T>G	ch	DD, diabetes, epilepsy, LA, cerebral atrophy	[6]
50 y	F	c.822G>C	hz	HA, ataxia, tremor, spasticity	[4]
49 y	M	c.822G>C	hz	HA, ID, epilepsy, behavioral disorder, dysphagia	[4]
47 y	F	c.822G>C	hz	HA	[4]
3 y	M	c.506T>A	hz	HA	[5]
2 m	F	c.185T>C, c.251+2T>G	ch	Epilepsy, hypotonia, BGL, HA	[3]
5 m	F	c.185T>C, c.509T>G	ch	Epilepsy, hypotonia, LA, cerebral atrophy	[3]
4.5 m	F	c.500A>G	hz	HA, hypotonia, myoclonic epilepsy, diabetes, DD, bleeding	[1]
3.5 y	F	c.182C>T, c.446A>AG	ch	DD, PMR, ID, epilepsy, spasticity	Index case

**Table 2:** Phenotypic features of NARS2-related mitochondrial disorders reported as of the end of July 2023

Organ/tissue	Feature	Reference
Central nervous system	Epilepsy	[10,11]
	Spasticity	[3,4,8,9]
	Intellectual disability	[10]
	Hypotonia	[11]
	Cortical blindness	[11]
	Psychomotor regression	[11]
	Ataxia	[15]
	Dystonia	[11]
	Chorea	[8]
	Optic atrophy	[11]
	Cortical/diffuse atrophy	[11]
	Hypomyelination	[11, index case]
	White matter lesions	[12]
	Subdural hematoma	[1]
	Agenesis of corpus callosum	[11]
Ears	Hypoacusis	[12]
Endocrine organs	Diabetes	[1,6]
	Growth retardation	[3,4]
Heart	Myocardial thickening	[11]
	Dilative cardiomyopathy	[11]
Guts	Vomiting	[11]
	Reflux	[11]
	Dysphagia	[11]
	Hepatopathy	[11]
Kidney	Tubulopathy	[11]
Muscle	Myopathy	[1,10]
Others	Lactic acidosis	[12]
	Elevated urine organic acids	[12]
	Microcephaly	[7,11]
	Opisthotonus	[8]
	Clubbed fingers	[15]
	Brachymetatarsalia	[15]
	Hallux	[15]

**Figure 1:**
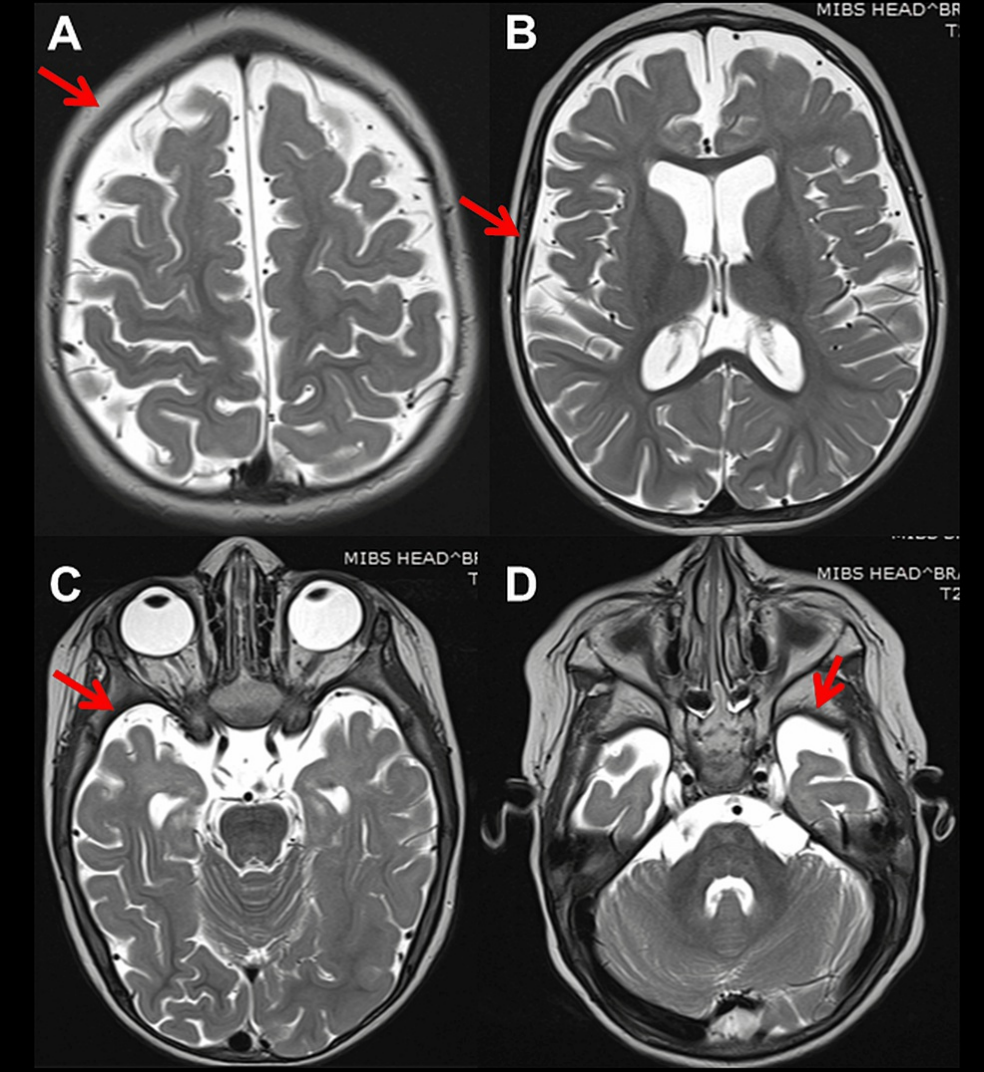
Cerebral MRI, T2-weighted images, at age 27 months showing hydrocephalus with dilation of lateral ventricles (B) due to reduced volume of the cortex (A), white matter, slight asymmetry of hippocampi (R > L), hyperintensities of the temporal poles bilaterally (C, D) and anterior parts of frontal lobes, smoothing of the cortico-medullary differentiation as a manifestation of incomplete myelination

**Figure 2:**
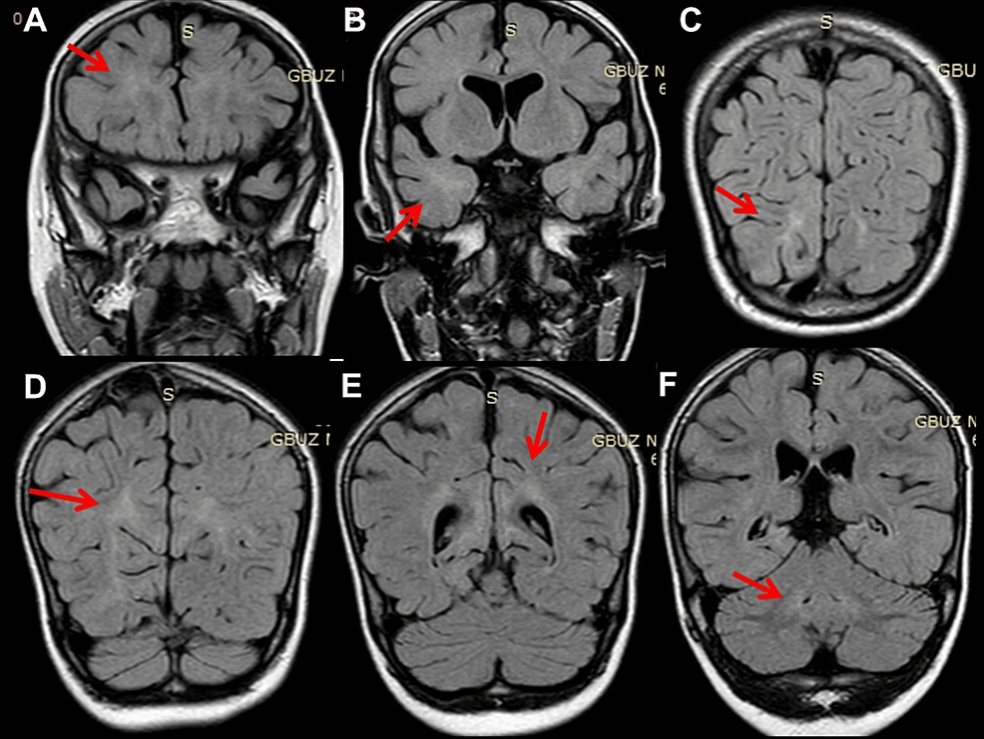
Cerebral MRI, fluid-attenuated inversion recovery (FLAIR) images, at age 27 months, showing hypomyelination supratentorially (A-C) and infratentorially (D-F), with right-sided predominance

At 12 months of age, she experienced a second SE, again terminated by DP, hydroxy-butyrate, and TP. VPA was switched to phenobarbital (PB). EEG at 15 months showed general slowing, delta activity across the occipital projections, and regional spikes along with delta bursts over O2-T6. At 16 months of age, she suffered a third SE, so PB was switched to oxcarbazepine (OXC), which was discontinued after seven days for ineffectiveness and replaced with perampanel (PER) in monotherapy (Table 3). EEG showed general slowing and focal seizure activity across F3-C3 associated with the eye version to the right. Post-ictal lethargy and decreased motivation occurred. Neurological evaluation after recovery revealed generalized hypotonia, discoordination, unstable eye contact, drooling, open mouth, and ankle contractions. She liked watching cartoons and playing with water. At 27 months of age, clonazepam (CZP) was added to PER with no beneficial effect (Table 3). Subsequently, ethosuximide (ESM) was added to PER but discontinued after 14 days due to ineffectiveness (Table 3). Cerebral magnetic resonance imaging (MRI) at 27 months of age showed bilateral diffuse cortical and subcortical atrophy (Figure 1) and incomplete myelination (Figure 2). At 33 months of age, levetiracetam (LEV) was tried but discontinued after four weeks because of ineffectiveness (Table 3). At 34 months of age, clobazam (CLB) was added to PER but stopped because daily seizures recurred. At 36 months of age, there was still no head control, but she had periodic torticollis to the left, tetraspasticity, increased tendon reflexes, positive pyramidal signs, and widespread hyperkinesia most pronounced in the left shoulder and unrelated to EEG activity. Topiramate (TPM) and hydrocortisone were started and PER was discontinued, stopping seizures for the next 40 months (Table 3). At the age of 40 months, she was switched to therapy with PER and TPM (Table 3).

**Table 3:** AED regimen since the onset of epilepsy at age 4.5 months until age 43 months AED: antiepileptic drug; CBD: cannabidiol; CLB: clobazam; CZP: clonazepam; DP: diazepam; ESM: ethosuximide; HB: hydroxybutyrate; LAC: lacosamide; LEV: levetiracetam; m: months; na: not applicable; OXC: oxcarbazepine; PB: phenobarbital; PER: perampanel; ps: persistent seizures; SE: status epilepticus; TP: thiopental; TPM: topiramate; VPA: valproic acid.

Age	Event	AED	Effect	Stopped at	Reason
4.5 m	1. SE	DP, TP, HB, VPA (240-360 mg/d)	7 months seizure free	2. SE	Ineffective
12 m	2. SE	DP, TP, HB, PB (6.25-25 mg/d)	ps	3. SE	Ineffective
16 m	ps	OXC (90 mg/d)	Ineffective	16 m	Daily seizures
17 m	3. SE	PER (1-4 mg/d)	1 seizure/month	Stopped 36-40 m	Ongoing 4 mg/d at 43 m
27 m	ps	CZP (0.5 mg/d)	ps	33 m	Depression
27 m	ps	ESM (100 mg/d)	Ineffective	28 m, after 2 w	Ineffective
33 m	ps	LEV (100 mg/d)	Ineffective	35 m	Seizure frequency
35 m	ps	CLB (2.5-10 mg/d)	Ineffective	34 m	Daily seizures
36 m	Daily seizures	Hydrocortisone (120 mg/d)	Myoclonus, seizures stop	40 m	Side effects
36 m	Daily seizures	TPM (12.5-90 mg/d)	Myoclonus, seizures stop	43 m	90 mg/d at age 41 m
43 m	Recurrence	LAC, CBD, Atkins diet	Partially effective	Ongoing	na

The transthoracic echocardiography and the long-term ECG recording were non-informative. Respirometry of skin fibroblasts was normal. Genetic workup using whole exome sequencing (WES) and Sanger sequencing at 12 months of age revealed the compound heterozygous variants chr11: 78204182C>T and chr11: 78282446A>AG in NARS2. She had inherited the variant chr11: 78204182C>T from her father and the variant chr11: 78282446A>AG from her mother. Both parents showed no clinical symptoms. The index patient was the only child and there was no consanguinity between parents. In addition to the current AED therapy (PER, TPM), she received a “mitochondrial cocktail” consisting of coenzyme-Q10 (50 mg/d), L-carnitine (500 mg/d), vitamin B2 (50 mg/d), vitamin-E (200 ME/d), pyrrolo-chinolin-chinon (0.075 mg/kg), and L-arginine (1 g/d), as of the end of January 2023. By the beginning of March 2023, the AED therapy was switched to cannabidiol (CBD) oil, hydrocortisone, and lacosamide (LAC) instead of TPM and the Atkins diet (Table 3).

## Discussion

The index patient is of interest for MID because of a novel compound heterozygous variant in NARS2. The variant manifested phenotypically with a non-syndromic MID affecting only the brain. The case differs from previously reported cases because of the novel mutation, exclusive brain manifestations, and the positive effect of VPA, steroids, and PER on seizure activity. The most prominent phenotypic features of previously reported NARS2-related disease included hearing loss, refractory seizures, neurodevelopmental delay (NDD), and myopathy (Table 1) [1]. Myoclonus, diabetes, spasticity, lactic acidosis, and microcephalus have been reported less frequently (Table 1) [1]. Some of the phenotypic features can occur in isolation, such as hearing loss or epilepsy [3-7]. With early onset in infancy, patients often suffer from growth retardation, intractable epilepsy, and hearing loss [3,4]. As the disease progresses, spastic paraplegia and neurodegeneration (Leigh syndrome) develop, ultimately leading to death [3,4,8,9]. Few cases have been reported worldwide, but 31 variants are known in NARS2 (Table 1) [3,4].

NARS2-related MID is usually an early-onset disease. Only one patient with a late onset was reported [10]. As of the end of July 2023, 35 patients with a NARS2 variant were reported (Table 1). The ages ranged from two months to 50 years, but there was definitively an early-onset and late-onset form of the disease. Eighteen were male and 16 were female (Table 1). These 35 patients carried 31 different NARS2 variants; 18 were homozygous and 18 had a compound heterozygous variant. The phenotypic presentation was very heterogeneous and ranged from isolated hearing loss to developmental delay, psychomotor regression, epilepsy, including absences and SE, hypotonia, cortical blindness, ataxia, dystonia, cardiomyopathy with heart failure, hepatopathy, and tubulopathy (Tables 1, 2). The outcome was also very different. Some patients died a few weeks after birth, while others, particularly mildly affected, survived into adulthood. A subdural hematoma, possibly due to unobserved falls during seizures, has been reported in some patients. Dysphagia due to pharyngeal dysfunction, vomiting, and reflux has been reported in some other patients (Table 1).

The index patient also presented with intractable epilepsy, myocloni, psychomotor regression, tetraspasticity, and torticollis. Epilepsy presented with generalized tonic-clonic seizures and focal seizures. Myocloni were not associated with epileptiform discharges. Various seizure types have been reported in NARS2-related disorders. These include focal, generalized, epileptic spasms, infantile spams, myoclonic seizures, absences, and SE [1]. EEG findings can range from background rhythm slowdown, focal or multifocal spikes/multiple spikes, and hypsarrhythmia [1]. Cerebral imaging may show hydrocephalus, cortical and subcortical atrophy, white matter lesions, basal ganglia lesions, or subdural hematoma [1]. The index patient benefited most from VPA, steroids, and PER. All other AEDs were ineffective or only temporarily effective.

The pathophysiological consequences of the detected compound heterozygous variants have not been studied in detail, but it is known from similar NARS2 defects that homozygous or compound heterozygous NARS2 variants result in reduced production of the enzyme, reduced import into the mitochondrion, reduced ligation of asparaginase to tRNA molecules, and a combined oxidative phosphorylation deficiency 24. Some of the NARS2 mutations (e.g., c251+2T>G) caused various splicing abnormalities and produced truncated proteins. Other mutations (e.g., c.185T>C and c.509T>G) reduced the binding free energy of the NARS2 protein dimer. Functional analysis of the intronic NARS2 deletion c.922-21_922-19del showed that the deletion caused splicing errors and resulted in exon-9 skipping in the mRNA.

## Conclusions

This case demonstrates that the novel compound heterozygous variant in NARS2 can phenotypically manifest only in the brain with epilepsy, developmental delay, hypotonia, myocloni, cerebral atrophy, and hypomyelination, followed by tetraspasticity and dystonia. Epilepsy can be treated most effectively with valproate, steroids, and perampanel.
